# Comparative serum lipid and immunohematological values among adult pulmonary tuberculosis and tuberculosis lymphadenitis cases and their association with sputum bacilli load and time to culture positivity in Northwestern Ethiopia

**DOI:** 10.1186/s12944-023-01821-3

**Published:** 2023-04-27

**Authors:** Daniel Mekonnen, Endalkachew Nibret, Abaineh Munshea, Awoke Derbie, Yohannes Zenebe, Aimro Tadese, Tigist Birku, Endalamaw Tesfa, Mulusew Alemneh Sinishaw, Hailu Getachew, Yosef Gashaw, Gizachew Yismaw, Mihiretu M. Kebede, Baye Gelaw

**Affiliations:** 1 Department of Medical Laboratory Sciences, School of Health Science, College of Medicine and Health Sciences, Bahir Dar University, Bahir Dar, Ethiopia; 2 Health Biotechnology Division, Institute of Biotechnology, Bahir Dar University, Bahir Dar, Ethiopia; 3 Department of Biology, Bahir Dar University, Bahir Dar, Ethiopia; 4 The Centre for Innovative Drug Development and Therapeutic Trials for Africa (CDT-Africa), Addis Ababa University, Addis Ababa, Ethiopia; 5 Amhara Public Health Institute, Bahir Dar, Ethiopia; 6 Department of Medical Biochemistry, College of Medicine and Health Sciences, Bahir Dar University, Bahir Dar, Ethiopia; 7 German Cancer Research Center, Im Neuenheimer Feld 280, 69120 Heidelberg, Germany; 8 Department of Medical Microbiology, School of Biomedical and Laboratory Sciences, College of Medicine and Health Sciences, University of Gondar, Gondar, Ethiopia

**Keywords:** Serum lipid, Immunohematological values, Pulmonary tuberculosis, Tuberculous lymphadenitis, Ethiopia

## Abstract

**Background:**

The serum lipid and immunohematological values of tuberculosis lymphadenitis (TBLN) patients is poorly documented relative to pulmonary tuberculosis (PTB) cases. Therefore, the aim of this study was to investigate the serum lipid and immunohematological values of patients with TBLN in comparison with PTB (PTB) patients.

**Methods:**

An institution-based comparative cross-sectional study was conducted in Northwest Ethiopia from March to December 2021. The study participants were bacteriologically confirmed PTB (n = 82) and TBLN (n = 94) cases with no known comorbidity and whose ages was greater than 18 years and with no current pregnancy. Independent sample t-test, one-way ANOVA, box plot, and correlation matrix were used to analyze the data.

**Results:**

The body mass index (BMI), CD4 + T cell count, and high-density lipoprotein-Cholesterol (HDL-C) values were significantly higher among TBLN cases compared with PTB cases. Additionally, the total white blood cell (WBC) count, hemoglobin (Hb), total Cholesterol (CHO) and creatinine (Cr) values were relatively higher among TBLN than PTB (P > 0.05). On the reverse, the platelet count and triacylglycerol (TAG) values were relatively higher among PTB than in TBLN cases. While the mean days of culture positivity were 11.6 days for TBLN, the mean days of culture positivity were 14.0 days for PTB. Anemia and serum lipid values showed no correlation with sputum bacilli load and time to culture positivity.

**Conclusion:**

Tuberculous lymphadenitis patients were well-endowed with serum lipid, immunological and nutritional status compared with PTB cases. Hence, the high incidence rate of TBLN in Ethiopia could not be explained by low peripheral immunohematological values, malnutrition, Anemia, and dyslipidemia. Further study for identifying the predictors for TBLN in Ethiopia is highly desirable.

## Introduction

Tuberculosis (TB) ravaged humankind throughout history [1, 2] and over one billion people have died of TB over the past 200 years alone [3]. In 2020 it became the second leading cause of death among infectious disease next to COVID-19. Currently, more than 1.3 million people died of TB every year [4]. Pulmonary TB (PTB) and TB lymphadenitis (TBLN) are the two most common forms of active TB globally [5, 6]. However, the global TB response strategy is not vigilant and robust towards EPTB forms such as TBLN. This is because EPTB has little role in TB transmission [7, 8]. However, evidences showed that EPTB such as TBLN has future impact on global TB control by serving as a source of reactivation TB [9].

As high as 47% of annual TB cases are TBLN in Ethiopia [10–14]. The factor(s) behind the disproportionately high incidence rate of TBLN in Ethiopia remains elusive. Towards excavating the risk factors, ethnicity, pathogen genotype variation, HIV co-infection [15–17], over diagnosis [18], spill over transmission of bovine TB [19] and homelessness [20] failed to demonstrate consistent association. Others such as being female, younger age[7, 17], delayed diagnosis, rural residency [21–24], chronic respiratory disease [25], end stage renal disease, immunosuppression [20], rheumatic diseases [26] and gene polymorphism in the pattern recognition receptors (PRR) [27] revealed significant association.

The factors that dissect PTB in to low (sputum smear negative and scanty) and high (sputum smear grade + 2 and + 3) *M. tuberculosis (Mtb)* bacilli load are not clearly outlined. Based on a study from Dakar, Senegal, absence of cavitation and cough, being HIV positive, having CD4+ T cell count above 200/mm^3^ and age over 40 years showed significant association with sputum smear negativity [28]. On the contrary, incarceration, sex, diabetes, alcohol dependence demonstrated significant association with smear positivity [29].

Host lipid is the main energy source and structural substrate for Mtb lipid metabolism [30, 31]. Hence we hypothesized that host serum lipid values would demonstrated correlation with forms of TB (PTB Vs TBLN), sputum bacilli load (low, medium and high) and Mtb time to culture positivity. Several lines of evidences showed apparent association between serum lipid value with TB [32–34], risk of TB [35], smear grading [32, 34], disease severity and inflammation [34].

Tuberculosis cases often became either leukopenia or leukocytosis [36] with neutrophilia [36, 37] and thrombocytosis being most common [38, 39]. High prevalence of anemia among TB patients and high rate of TB among people with different forms of anemia have been reported by different studies [40–42]. However, whether anemia is a predisposing factor for TB or the vice versa remains an open question [41, 42]. The mean CD4 + T cell value among PTB and disseminated TB was significantly lower when compared with TBLN (p < 0.05) [43]. These findings suggest that different clinical forms of TB might have distinct profiles of peripheral blood markers. In general, few studies so far assessed the immunohematological and serum lipid profile of TBLN cases relative to PTB cases in Ethiopian context.

Hence, the aim of this study was to compare the immunohematological and serum lipid values between PTB and TBLN cases and then assess the correlation between sputum bacilli load and Mtb time to culture positivity with serum lipid values.

## Methods

### Study design, period and setting

An institution-based comparative cross-sectional study was conducted in Bahir Dar, Northwestern Ethiopia from March to December 2021. There are 10 public health centers and three governmental hospitals in Bahir Dar city administration. The data were collected from four of these health facilities namely Felege Hiwot Comprehesive Specilized Hospital (FHCSH), Han, Bahir Dar and Shum Abo health centers. The four health facilities were selected based on patient load and availability of data collector. No sample size allocation was done to each health facility.

Bahir Dar was established in the first half of 13th century. It became the capital city of the Amhara Regional State since 1993. Bahir Dar is the heart of Blue Nile River and located 578 km away from Addis Ababa, the capital city of Ethiopia. The city is located at an elevation of 1808.01 m above sea level. The city has a temperate highland tropical climate with an average yearly temperature of 22.26ºC. The population of Bahir Dar is estimated to be 455,901. Of whom 228,189 were male and 227,712 were female (Projection for 2022-07-01). It was also estimated that 81.2% of the population are urban inhabitants and the rest are living in rural kebeles around Bahir Dar [44, 45].

### Participants

Despite, the study was conducted in Bahir Dar, the source population included surrounding zones (South and North Gondar, East and West Gojjam and Awi). The study participants were bacteriologically confirmed PTB (N = 82) and TBLN (N = 94) cases. The PTB cases were bacteriologically confirmed by GeneXpert and/or Microcopy. The TBLN cases were initially diagnosed with cytology and then confirmed with culture or Ziehl-Neelsen (ZN) staining techniques. The TBLN cases who were diagnosed by cytology but were negative by bacteriological techniques were excluded. Among those bacteriologically confirmed cases, subjects whose age less than 18 years, with comorbidity such as HIV or diabetes mellitus and with current pregnancy were excluded from the study. Patients who had both PTB and TBLN were also excluded. The instruments and enrolment procedures were the same throughout the study and, the study settings were similar for all TB patients.

### Sample size and sampling technique

**Fig. 1:**
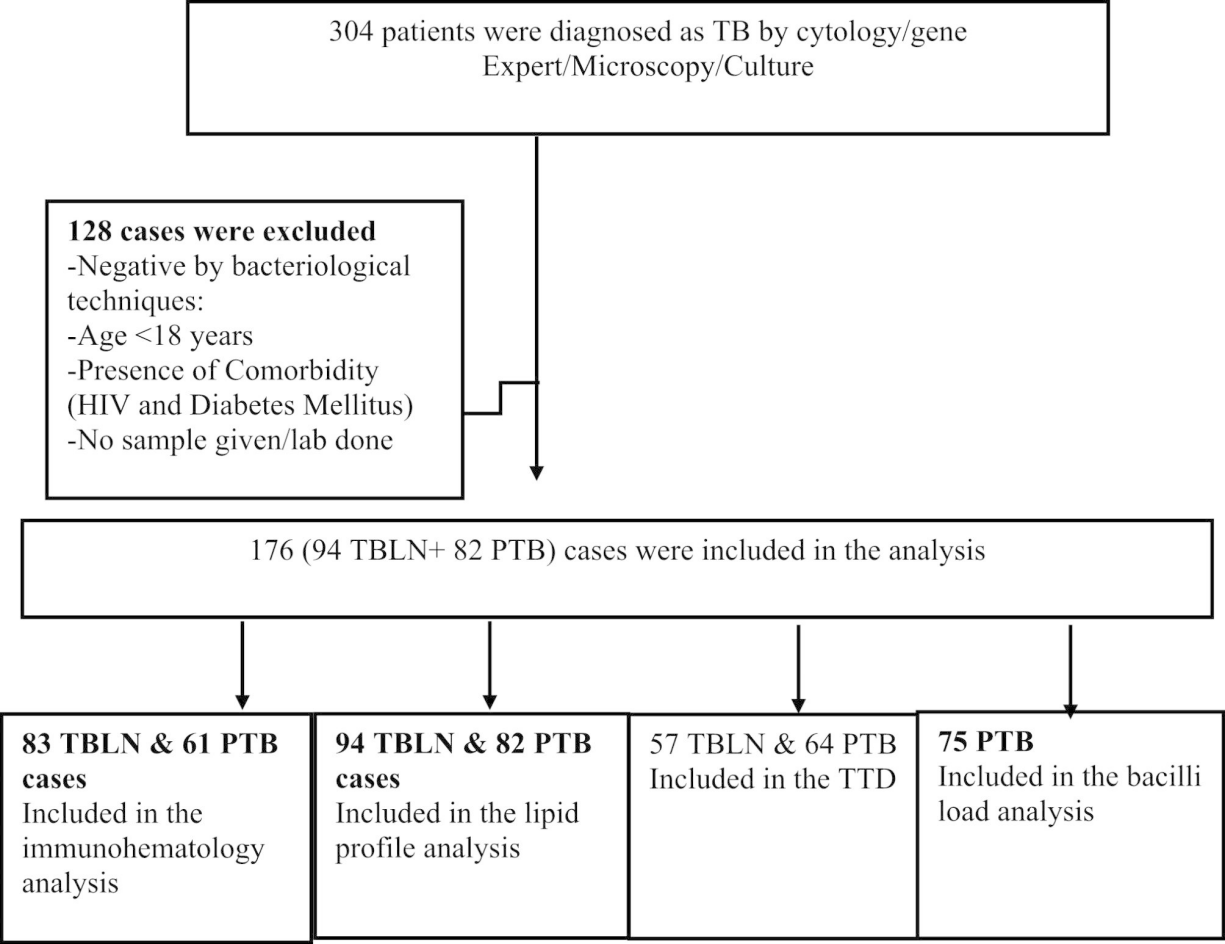
Study participant enrolment and selection strategy, Northwest Ethiopia, 2023

The sample size was determined using G*Power v3.1.9.4 statistical power analyses tool. The estimation considered the mean difference of two independent sample T test with effect size 0.5, α error probability of 5%, 95% power (β) and allocation ratio of 1. Substituting these, it gave a total of 176 sample size. To achieve this, newly diagnosed cases were consecutively invited to be included in the study. A total of 200 TBLN and 104 PTB cases were identified and screened (Fig. 1).

### Laboratory methods

Two to five milliliters (ml) of sputum and 6 ml of venous blood were collected from bacteriologically confirmed PTB cases. From cytological diagnosed TBLN cases, another fine needle aspiration cytology (FNAC) sample and 6 ml of venous blood were obtained. The FNAC samples were further confirmed by ZN microscopy and culture at Amhara Public Health Institute (APHI) before enrolment in the final analysis. Sputum bacilli load was measured using ZN acid fast bacilli (AFB) staining technique. The Mtb time to culture detection/positivity (TTD) was measured using Mycobacterium growth indicator tube (MGIT)-960 technique.

The collected blood sample from each participant was aliquoted into EDTA and serum separator tubes, each 3ml. The EDTA tube was shipped to APHI or FHCSH immunohematology laboratory for complete blood count and CD4 + T cell count. The hematological variables were measured using five part (BC-5800 Mindray, Shenzhen Mindray Bio-Medical Electronics Co., Ltd) or three part (sysmex xs-500i) auto hematology analyzer. The absolute CD4 + T cell count was measured at APHI using BD FACSPresto™ system which has over 96% agreement with gold standard methods (BD FACS Calibur and sysmex systems) [46].

The serum samples were separated and stored at -80^o^c. Serum concentrations of total cholesterol (CHO), triglyceride (TAG), HDL-cholesterol (HDL-C) and creatinine (Cr) were determined by the closed system Dimension EXL 200 Integrated chemistry analyzer at Tibebe Ghion Comprehensive Specialized Hospital. We used the products of Siemens Healthineers reagents for the determination of CHO, TAG, HDL-C and Cr. Total cholesterol was determined by cholesterol oxidase- horseradish peroxidase methods with the analytical sensitivity of 1.295 mmol/L (0.0259*50 mg/dL) and 1.295 − 15.54 mmol/L measurement ranges while TAG was determined by glycerol-3-phosphate-oxidase-peroxidase technique with an analytical sensitivity of 0.1695 mmol/L (0.0113*15 mg/dL) and 0.1695–11.3mmol/L measurement ranges. HDL-cholesterol was determined by the modified cholesterol esterase and cholesterol oxidase methods with the analytical sensitivity of 0.0777mmol/L(0.0259*3 mg/dL) and 0.0777–3.885mmol/L measurement ranges while creatinine values was measured by the modified Jaffe technique with an analytical sensitivity of 13.26µmol/L (88.4*0.15 mg/dL) and 13.26–1768 µmol/L measurement ranges. The tests were performed in batches according to the manufacturers’ instruction. Before performing the laboratory analysis, the machine was calibrated and validated with standards. In addition internal quality control was performed to maintain the quality of generated data. The analysis was performed using the laboratory standard operating procedure.

### Variables

The study variables include age, sex and body mass index (BMI) in kilogram per meter squared (Kg/m^2^), white blood cells count (WBC*10^9^/L), platelet count (PLT*10^9^/L), the CD4 + T cell count (CD4 + cell*10^6^/L), hemoglobin (Hb g/L), Cholesterol (CHO mm/L), triacylglycerol (TAG mm/L), high density lipoprotein-Cholesterol (HDL-C mm/L) and creatinine (Cr µmol/L).

The BMI was calculated using the formula, weight in Kg/height in M^2^. The BMI were classified based on Nuttall 2015 [47] and WHO classification [48]. Accordingly, BMI (Kg/m^2^) < 18.5 is classified as underweight (UW), BMI (Kg/m^2^) = 18.5–24.9 (Normal), BMI (Kg/m^2^) = 25-29.9 (Over weight/pre obese) and BMI (Kg/m^2^) > 30 (Obese).

The Hb values was used to classify anemia based on the WHO recommended ranges for adult. As such, anemia was classified into three categories: mild (110–119 g/L) for non-pregnant women and (110–129 g/L) for men, moderate (80–109 g/L) and severe (< 80.0 g/L)[49].

### Ethical considerations

The study was approved by research and ethical review committee of Science College of Bahir Dar University, with reference number PGRCSV/111/2012. Informed written consent was obtained from each participant before data collection. This study was conducted in accordance with the Declaration of Helsinki. All the information obtained from the study subjects were coded to maintain confidentially.

### Bias and quality assurance system

The immunohematological and serum lipid analyses were conducted in laboratories which have standardized quality assurance system and were under continuous external quality assurance system. There were daily quality control activities using three level (low, normal and high) commercial quality control (QC) materials. Then, the analyzers were calibrated as per the manufacturer recommendations.

### Statistical analyses

Chi square test was used for summarizing categorical variables. The mean with standard deviation (SD) and median were used for describing the continuous variables. Normality was assessed using Kolmogorov-Smirnov (K-S) and Shapiro-Wilk tests. Independent sample T test with 2 sided P-value was used to compare mean difference between PTB and TBLN with regard to the included immunohematological and serum lipid parameters. One way ANOVA with post hoc pair wise multiple comparison test was computed to determine the correlation between sputum bacilli load (low, medium and high) with serum lipid and other continuous variables. Box-and-Whisker plots were created for depicting the correlation of sputum bacilli load with TTD, TAG, CHO and HDL values. The relationship between time to Mtb detection and serum lipid value was assessed using Pearson Correlation (r) and scatter plot matrix. The overall analyses were carried out using R version.4.0.4 (*R Core Team 2021), URL*https://www.Rproject.org/*)* and SPSS version 25 (*IBM Corp. Released 2017. IBM SPSS Statistics for Windows, Version 25.0. Armonk, NY: IBM Corp*.).

## Results

### Characteristics of participants

Among the total 176 TB patients, 94 (53.4%) were TBLN and 82 (46.6%) were PTB. Of the total 176 TB study participants, 100 (56.8%) were males. The mean ages (SD) were 33.7 (14.2) and 32.2 (12.2) years for TBLN and PTB cases, respectively. There were no significant difference in terms of age between PTB and TBLN cases (P = 0.466). Among the total 176 TB patients, male PTB accounted 53 (30.1%) and female TBLN accounted 44 (26.7%). When classifying total female TB patients into TBLN and PTB, 61.8% were TBLN (X^2^ = 3.82, P = 0.05). The Kolmogorov-Smirnov test P-value and visual inspection of the histogram showed that, the data were roughly normally distributed.

### Comparative lipid, immunohematological and BMI values between TBLN and PTB

Independent sample T- test and Mann Whitney U tests were performed for comparing mean and median difference, respectively between TBLN and PTB cases. The overall statistical results were concordant between the two models. Hence, in cases of the p-values of Levene’s test > 0.05 (age, WBC, CD4 cell count, BMI, Cr and TTD), the P value of t-test with equal variance assumed was considered and otherwise, P-values for equal variance not assumed was used for comparing means between the groups. The mean, median, SD and independent sample T test statistics are summarized below (Table 1).

**Table 1:** Mean and median values with SD of age, BMI, immunohematological, serum lipid and creatinine values of PTB and TBLN patients in Northwestern Ethiopia, 2023

Dependent Variables	TBLN				PTB				P-Value
	N	Mean	Median	SD	N	Mean	Median	SD	
Age (Year)	94	33.7	30.0	14.2	82	32.2	29.0	12.2	0.466
BMI (Kg/m2)	94	18.9	18.7	2.2	82	17.3	17.2	2.7	**0.001**
WBC*10^9^/L	83	7.4	7.4	2.1	61	7.2	6.9	2.6	0.534
Hb (g/L)	83	126.6	130.0	19.7	61	120.6	126.0	27.7	0.152
PLT*10^9^/L	83	301.04	292.0	101.8	60	315.9	308.0	131.0	0.463
CD4 cell*10^6^/L	40	759.0	792.5	294.3	48	456.	400.5	279.0	**0.001**
CHO (mmol/L)	94	2.5	2.7	1.4	82	2.4	2.4	0.9	0.749
TAG (mmol/L)	94	0.7	0.7	0.5	82	0.85	0.8	0.4	0.065
HDL-C (mmol/L)	94	0.8	0.9	0.4	82	0.6	0.6	0.3	**0.019**
Cr (µmol/L)	94	73.3	66.7	124.4	82	71.6	72.9	19.8	0.902
Time to grow (days)	57	11.6	11.1	6.0	64	14.0	8.7	13.0	0.194

***BMI***: *body mass index*, ***TB***: *Tuberculosis*, ***WBC***: *White blood cell count*, ***Hb***: *Hemoglobin*, ***PLT***: *Platelet*, ***N***: *number of cases*, ***TBLN***: *tuberculous lymphadenitis*, ***PTB***: *Pulmonary tuberculosis*, ***CHO***: *Cholesterol*, ***TAG***: *triacylglycerol*, ***HDL***: *High density lipoprotein*, ***UW***: *Underweight*, ***SD***: *Standard deviation*

***Normal values for***: ***BMI (Kg/m2)***: *18.5–24.9*[47, 50]. WBC*10^***9***^***/L***: *4.8–10.8*[51]. ***Hb (g/L)***: *non-pregnant women (15 years of age and above): >120 g/L, Men (15 years of age and above):>130 g/L*[49]. PLT*10^***9***^***/L***: *150–400*. CD4 + Cells*10^***6***^***/L***: *396–1598* [52]. ***CHO (mmol/L)*** : *<5.17 is normal, 5.17–6.18 is high, ≥ 6.21 is high.****TAG (mmol/L)***: *normal: <1.7, mildly increased: 1.7–5.6, moderately increased: 5.6 to 10.0, Very high: >10.0. .****HDL-C (mmol/L)***: *≥1.55 excellent, < 1.03 is considered lower than desirable* [53]. ***Cr (µmol/L)***: *61.9 to 114.9 µmol/L for men and 53 to 97.2 µmol/L for women* [54, 55].

Table 1 shows that, there is statistically significant difference between TBLN and PTB in terms of CD4 + T cell count, BMI, HDL-C values. While immunohematological values of both groups of patients fall within normal range, the lipid profile is variable when comparing with normal reference range. For instance, total CHO and TAG values are within the normal range and HDL-C which is the good part of cholesterol is below the normal range. The mean BMI value of PTB patients is below the reference limit.

### Correlation of sputum bacilli load with serum lipid values, Hb and TTD

**Fig. 2:**
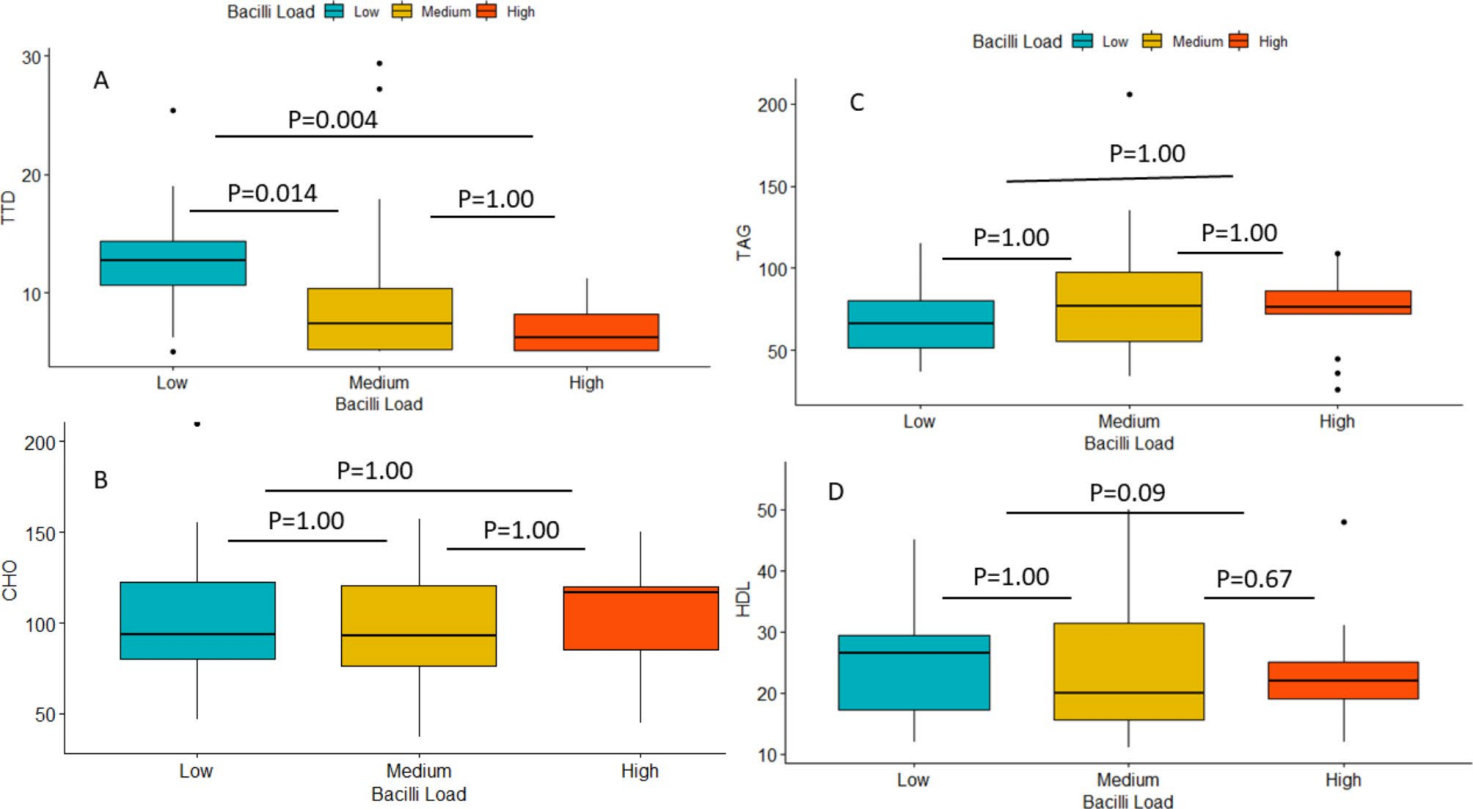
Box and whisker plot showing the correlation between sputum bacilli load with serum lipid values and time to Mtb culture positivity, Northwestern Ethiopia, 2023. (*Box and whisker plot depicted the minimum score, the lower quartile (25%), the median (50%, horizontal line), upper quartile (75%), maximum values and outliners (black dots).****Low***: *sputum smear negative and scanty*, ***Medium***: *+1*, ***High***: *+2 & +3*)

Studies showed that the Mtb bacilli load was very low in FNA samples [56, 57] including the present study. Hence, FNA samples were excluded and bacilli load was enumerated only for PTB samples. Based on box and whisker plot depicted in Fig. 2 below, the sputum bacilli load showed significant correlation with TTD in MGIT liquid culture. The median TAG and CHO values are slightly higher among patients with high bacilli load compared with PTB patients with low sputum bacilli load (Fig. 2B & C). The HDL-C (good cholesterol) value was high among TB patients with low bacilli load (Fig. 2D). However, none of the lipid profiles (CHO, TAG, and HDL-C) demonstrated significant association with sputum bacilli load (Fig. 2).

Anemia (mild, moderate and severe) was more prevalent in PTB (30/61, 49.2%) than in TBLN cases (31/83, 37.3%), Pearson χ^2^ = 2.02, p = 0.156. Exploring anemia among female and male TB patients revealed that, the prevalence of anemia (mild, moderate and severe) was 43.1% and 42.3% among female and male TB patients, respectively (Pearson χ^2^ = 0.75, p= 0.688). Out of the total 29 anemic and 30 non anemic TB patients, only 7 (24.1%) and 7 (23.3%) had high bacilli load in their sputum. The Chi square (χ^2^ = 0.2) test and one way ANOVA revealed absence of correlation between anemia and bacilli load (p=0.89).

### The correlation between times to Mtb detection with host serum lipid values

**Fig. 3:**
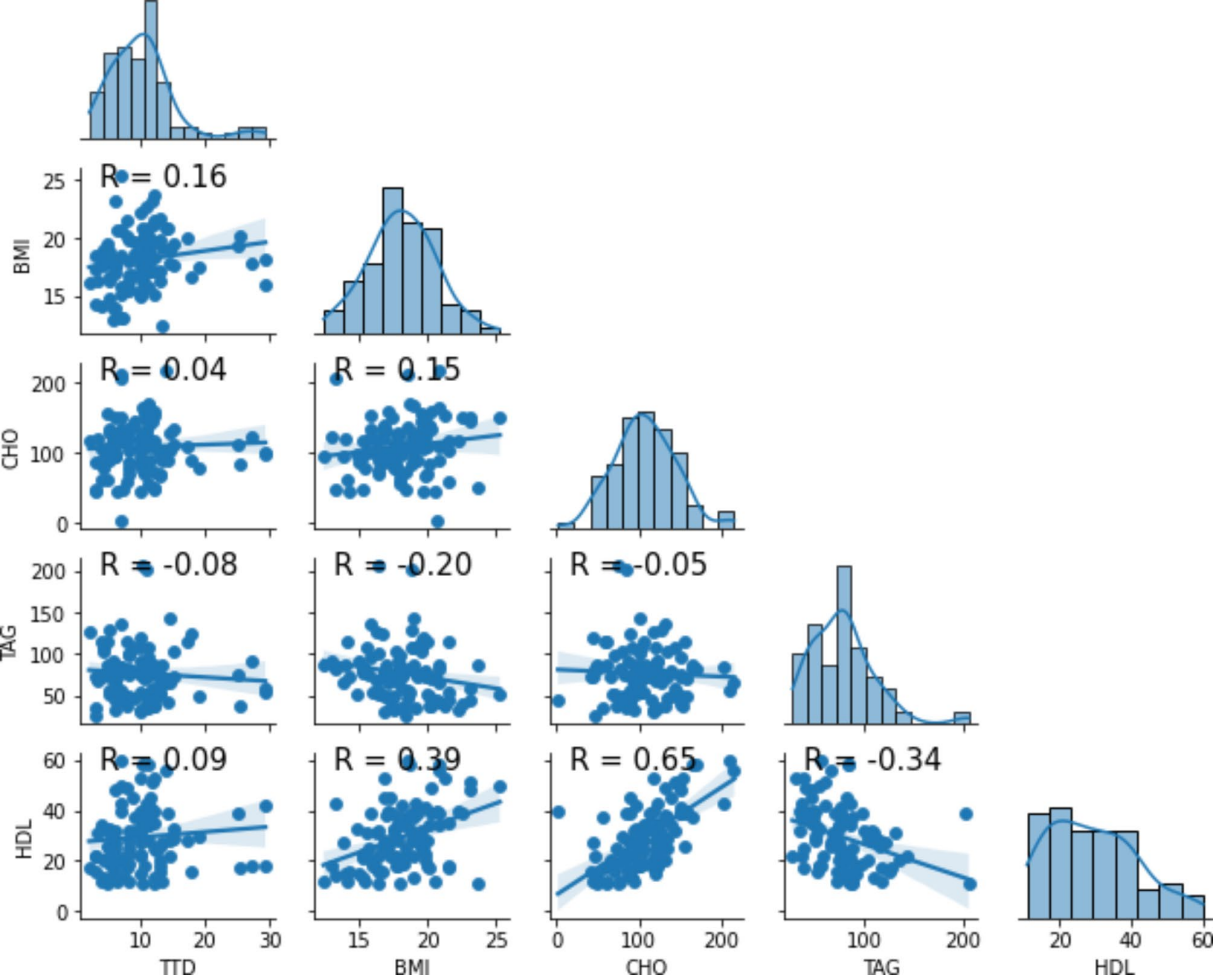
Scatter plot matrix showing the correlation between times to Mtb detection with BMI, serum lipid and creatinine values 2023. (***TTD***: *time to detection/culture positivity*, ***BMI***: *Body mass index*, ***CHO***: *Cholesterol*, ***TAG***: *triacylglycerol*, ***HDL***: *High density lipoprotein*, ***Cr***: *Creatinine*)

In the TTD analysis, Pearson correlation and scatter plot matrix (Fig. 3) were used for the evaluation. Body mass index showed statistically non-significant positive correlation with TTD. Patients with higher values of BMI (good nutritional status), tend to have delayed culture positivity (r = 0.16) (Fig. 3). As shown in Fig. 3 below, no significant correlation was found between TTD and serum lipid values (r ~ 0.0, p > 0.05).

## Discussion

The purpose of this study was to investigate mean differences in serum lipid, selected immunohematological, BMI and creatinine values between TBLN and PTB and further confirm the correlation between serum lipid values with time to Mtb culture positivity and sputum bacilli load. Hence, the evaluation provided important metabolic, nutritional and clinical information about TBLN and PTB cases.

The results in Table 1 indicated a significant mean difference between TBLN and PTB with regard to BMI, HDL-C and CD4 + cells; all being higher among TBLN than PTB. The mean total WBC and Hb values were also relatively higher among TBLN than PTB cases. The higher values of all these variables imply better nutritional, metabolic and immunological status for TBLN compared with PTB [58, 59]. In line with this study, a study in Jimma University Hospital found lower mean CD4 + cells count and Hb value among PTB cases compared with TBLN [60]. Patients with disseminated TB showed significantly lower CD4 cell counts compared with those with localized form of TB in the lymph nodes [61].These results provided further support for the hypothesis that unlike disseminated TB which is characterized by lower values of peripheral blood immunohematological parameters relative to the PTB, TBLN in Ethiopian could be characterized as a unique form of TB, with higher mean values of WBC and CD4 + T cell counts relative to PTB. However, the sample size of our study and the study carried out in Jimma [61] were small which precluded us from making strong conclusion.

High-density lipoprotein is involved in lipid metabolism, organism’s immune and antioxidant defense and has a role in transport and in the removal of exogenous substances [62]. Unfortunately, the value of this good lipid is below the normal range for both groups of TB patients. This might be due to the association of HDL-C with Mtb Lipoarabinomannan (LAM) nanodiscs in human serum [63]. Higher serum CHO levels were associated with lower mortality and considered as a marker of lower levels of inflammation in TB patients and this condition was unaffected by BMI [64]. On the contrary, low serum CHO in PTB cases might be due to high oxidative stress and elevated cytokines [32].

The peripheral blood platelet count was marginally higher in PTB than in TBLN cases with no significant difference. Evidence showed that TB is associated with thrombocytosis and this condition is correlated with disease severity [38] and Mtb smear positivity [65]. Unlike Renshaw and Gould (2013) [65] study, the present study did not identify any correlation between platelet count and sputum smear bacilli load. Based on the PLT counts value, it can confirmed that the degree of inflammation in TBLN was relatively mild compared with PTB; hence lower disease severity. Platelets mainly drive TB immunopathology on their effect on other immune cells, chiefly monocytes and neutrophils. Like innate immune cells, platelets also participate in the initial detection of Mtb through their PRR. Moreover, platelet also participate in inflammatory process through the release of Reactive Oxygen Species (ROS), pro-inflammatory cytokines, activation of monocyte and neutrophil effector functions [38].

Anemia was more prevalent in PTB (49.2%) than in TBLN cases (37.3%), Pearson χ^2^ = 2.02, p = 0.156. Exploring anemia among female and male TB patients revealed prevalence of 43.1% and 42.3% among female and male TB patients, respectively (earson χ^2^ = 0.75, p = 0.688). Similarly, previous reports revealed a much higher proportion of anemia among TB patients [66–68]. Anemia is potentially a risk factor for TB disease severity [67, 68] and delayed smear conversion [69]. However, whether TB or anemia is the risk factor is largely unknown. Anemia did not showed any correlation with sputum bacilli load in the present study.

The lipid profiles (CHO, TAG, and HDL) and immunohematological values failed to demonstrated significant association with sputum bacilli load in the present study. Unlike this study, studies done elsewhere, reported a negative correlation between serum lipid values and sputum bacilli load [32, 34, 70]. This negative correlation might be due to high oxidative stress and elevated cytokines [32].

**Fig. 4:**
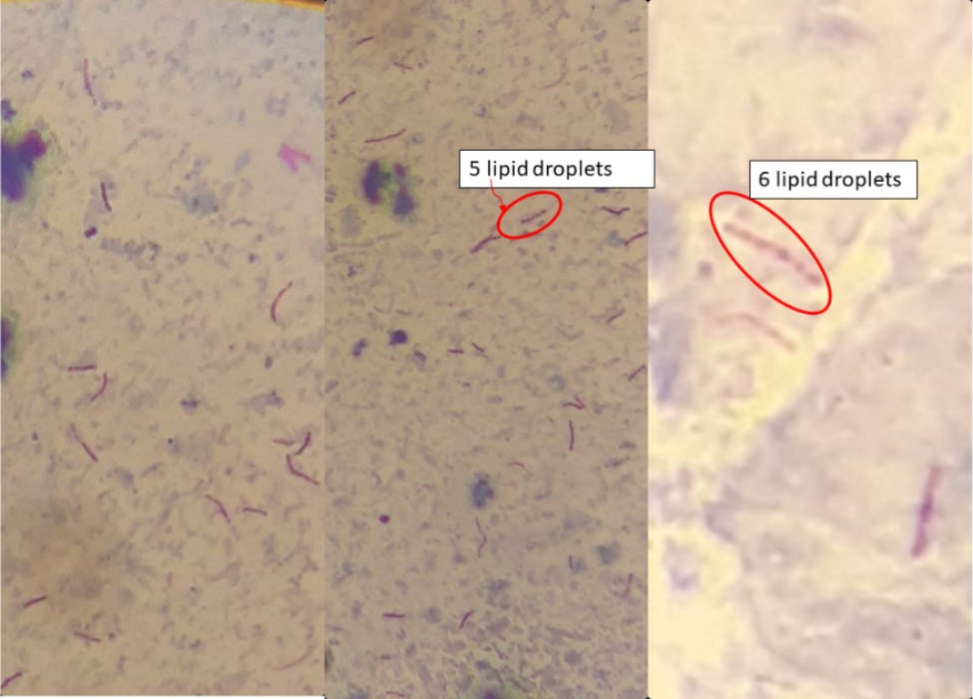
Mycobacterium tuberculosis lipid droplets in dual ZN-Sudan black B staining, Northwest Ethiopia, 2023

Significant negative correlation was observed between TTD and sputum Mtb load (Fig. 2A). Further attempt was made to measure correlation of TTD with bacilli lipid body count/lipid droples (Fig. 4). However, the Sudan black B staining technique became imprecise for accurate enumeration of the lipid droplets and excluded. As shown in Fig. 3, the correlation between time to Mtb culture positivity with serum lipid values was trivial (r ~ 0, p > 0.05). Host lipids act as the major source of carbon and energy for Mtb. Fatty acids derived from the host cells are converted to TAG and stored in the bacterial cytoplasm [71].

The mean TTD was 11.6 days for TBLN isolates and 14.0 days for PTB isolates. However, the difference was not statistically significant (p = 0.194). The relatively delayed positivity for sputum isolates relative to FNA isolates might be due to the organic load present in the sputum sample and the presence high number of lipid droplet positive Mtb in sputum samples [72]. Additionally, sputum samples were decontaminated with NALC-NaOH while FNA samples were inoculated directly without pretreatment. This sample treatment step might weaken the viability of sputum Mtb and took longer lag phase than FNA originated Mtb isolates.

### Limitations

This study determined the mean serum lipid, Cr, BMI and selected peripheral blood immunohematological values among TBLN cases in comparison with PTB. The study has some methodological limitations. Despite the data collection was carried out at four health facilities, the study was not multicenter; not from different geographic locations to make better comparison. The sample size from each study group was small. The use of two hematology analyzer might not a good practice. Lipid profile might be affected with meal and the current study was done using random sample rather than fasting sample. The population were not stratified with urban versus rural. Despite that, our analysis was detailed to validate the analysis output. Hence, this finding would be an important addition to the existing and future literature to better understand the risk factors responsible for the higher incidence rate of TBLN in Ethiopia.

## Conclusions and recommendations

Except the PLT and TAG values whose values are relatively lower among TBLN, the immunohematological variables such as WBC (p > 0.05), Hb (p > 0.05), CD4 + cells (p < 0.05) and the serum lipid values; CHO (P > 0.05), HDL-C (P < 0.05) and Cr (p > 0.05) values were relatively higher among TBLN than PTB. The mean TTD was 11.6 days for TBLN isolates and 14.0 days for PTB isolates. Serum lipid values and anemia have no any correlation with sputum bacilli load and TTD. The relatively higher values of WBC, CD4 + T cells, Hb and BMI and lower PLT count among TBLN confirmed the unique immunological, inflammatory, nutritional and “clinical evolution” of TBLN. Further research involving healthy controls, with multicenter study design, using probablity sampling strategy and using large sample size with fasting blood sample is very desirable.

## Data Availability

Almost all data generated and analyzed data during this study were included in the manuscript. But if the spreadsheet dataset is needed, it will be shared upon request by the editor from the corresponding author.

## Abbreviations

APHI: Amhara public health institute
BMI: Body mass index
CHO: Cholesterol
Cr: Creatinine
EPTB: Extra pulmonary TB
FHCRH: Felege Hiwot Comprehensive Specialized Hospital
FNA: Fine needle aspiration
FNAC: Fine needle aspiration cytology
Hb: Hemoglobin
HDL: High density lipoprotein
LJ: Löwenstein–Jensen
*M. tuberculosis Mtb*: *Mycobacterium tuberculosis*
MGIT: mycobacteria growth indicator tube
PLT: Platelet
PRR: Pattern recognition receptors
PTB: Pulmonary TB
QC: Quality control
TAG: Triacylglycerol
TB: Tuberculosis
TBLN: Tuberculous lymphadenitis
TGCSH: Tibebe Ghion Comprehensive Specialized Hospital
TLR: Toll like receptors
TTD: Time to detection
WBC: Total white blood cell
ZN: Ziehl-Neelsen

## References

[1] Daniel TM, Bates JH, Downes KA. History of tuberculosis. Tuberculosis: pathogenesis protection and control 1994:13–24.

[2] Barberis I, Bragazzi NL, Galluzzo L, Martini M (2017). The history of tuberculosis: from the first historical records to the isolation of Koch’s bacillus. J Prev Med Hyg.

[3] Gagneux S (2018). Ecology and evolution of Mycobacterium tuberculosis. Nat Rev Microbiol.

[4] WHO. : Global tuberculosis report In. Geneva; 2021.

[5] Golden MP, Vikram HR (2005). Extrapulmonary tuberculosis: an overview. Am Fam Physician.

[6] Gopalaswamy R, Dusthackeer VA, Kannayan S, Subbian S (2021). Extrapulmonary tuberculosis—an update on the diagnosis, treatment and drug resistance. J Respir.

[7] Varghese B, Al-Hajoj S. Mapping the epidemiology and trends of extra-pulmonary tuberculosis in Saudi Arabia. Int J Mycobacteriol *2015*, 4(4):261–9. 10.1016/j.ijmyco.2015.06.002 26964806

[8] Katsnelson A (2017). Beyond the breath: exploring sex differences in tuberculosis outside the lungs. Nat Med.

[9] Ganchua SKC, White AG, Klein EC, Flynn JL (2020). Lymph nodes—the neglected battlefield in tuberculosis. PLoS pathog.

[10] Alene KA, Clements AC (2019). Spatial clustering of notified tuberculosis in Ethiopia: a nationwide study. PLoS ONE.

[11] Tessema B, Muche A, Bekele A, Reissig D, Emmrich F, Sack U (2009). Treatment outcome of tuberculosis patients at Gondar University Teaching Hospital, Northwest Ethiopia. A five-year retrospective study. BMC Public Health.

[12] Mekonnen D, Derbie A, Mekonnen H, Zenebe Y (2016). Profile and treatment outcomes of patients with tuberculosis in northeastern Ethiopia: a cross sectional study. Afr Health Sci.

[13] Zenebe Y, Adem Y, Mekonnen D, Derbie A, Bereded F, Bantie M (2016). Profile of tuberculosis and its response to anti-TB drugs among tuberculosis patients treated under the TB control programme at Felege-Hiwot Referral Hospital, Ethiopia. BMC Public Health.

[14] Mekonnen D, Munshae A, Nibret E, Derbie A, Abeje A, Feleke BE et al. Tuberculosis case notification rate mapping in Amhara Regional State, Ethiopia: Four years retrospective study: Tuberculosis. *EMJ* 2022, 60(01).

[15] Berg S, Schelling E, Hailu E, Firdessa R, Gumi B, Erenso G (2015). Investigation of the high rates of extrapulmonary tuberculosis in Ethiopia reveals no single driving factor and minimal evidence for zoonotic transmission of Mycobacterium bovis infection. BMC Infect Dis.

[16] Firdessa R, Berg S, Hailu E, Schelling E, Gumi B, Erenso G (2013). Mycobacterial lineages causing pulmonary and extrapulmonary tuberculosis, Ethiopia. Emerg Infect Dis.

[17] Mekonnen D, Derbie A, Abeje A, Shumet A, Nibret E, Biadglegne F (2019). Epidemiology of tuberculous lymphadenitis in Africa: a systematic review and meta-analysis. PLoS ONE.

[18] Iwnetu R, Van Den Hombergh J, Woldeamanuel Y, Asfaw M, Gebrekirstos C, Negussie Y (2009). Is tuberculous lymphadenitis over-diagnosed in Ethiopia? Comparative performance of diagnostic tests for mycobacterial lymphadenitis in a high-burden country. Scand J Infect Dis.

[19] Ameni G, Vordermeier M, Firdessa R, Aseffa A, Hewinson G, Gordon SV (2011). Mycobacterium tuberculosis infection in grazing cattle in central Ethiopia. Vet J.

[20] Qian X, Nguyen DT, Lyu J, Albers AE, Bi X, Graviss EA (2018). Risk factors for extrapulmonary dissemination of tuberculosis and associated mortality during treatment for extrapulmonary tuberculosis. Emerg Microbes Infect.

[21] Asres A, Jerene D, Deressa W (2019). Delays to anti-tuberculosis treatment intiation among cases on directly observed treatment short course in districts of southwestern Ethiopia: a cross sectional study. BMC Infect Dis.

[22] Awoke N, Dulo B, Wudneh F. Total delay in treatment of tuberculosis and associated factors among new pulmonary TB patients in selected health facilities of Gedeo zone, southern Ethiopia, 2017/18. *Interdiscip Perspect Infect Dis* 2019, 2019. 10.1155/2019/2154240 PMC6582841 31275370

[23] Bilchut AH, Mekonnen AG, Assen TA (2022). Knowledge of symptoms and delays in diagnosis of extrapulmonary tuberculosis patients in North Shewa zone, Ethiopia. PLoS ONE.

[24] Shiferaw MB, Zegeye AM (2019). Delay in tuberculosis diagnosis and treatment in Amhara state, Ethiopia. BMC Health Serv Res.

[25] Dakheel MA, Najaim AM, Moslah AM, Buni HD (2021). The disseminated tuberculosis with cavitary lung lesion and tuberculoma in a six-month-old libyan infant. J Infect Dev Ctries.

[26] Lu M, Lai C, Tsai C, Koo M, Lai N (2015). Increased risk of pulmonary and extra-pulmonary tuberculosis in patients with rheumatic diseases. IJTLD.

[27] Abate E, Blomgran R, Verma D, Lerm M, Fredrikson M, Belayneh M (2019). Polymorphisms in CARD8 and NLRP3 are associated with extrapulmonary TB and poor clinical outcome in active TB in Ethiopia. Sci Rep.

[28] Samb B, Sow P, Kony S, Maynart-Badiane M, Diouf G, Cissokho S (1999). Risk factors for negative sputum acid-fast bacilli smears in pulmonary tuberculosis: results from Dakar, Senegal, a city with low HIV seroprevalence. IJTLD.

[29] Hermosilla S, You P, Aifah A, Abildayev T, Akilzhanova A, Kozhamkulov U (2017). Identifying risk factors associated with smear positivity of pulmonary tuberculosis in Kazakhstan. PLoS ONE.

[30] Maurya RK, Bharti S, Krishnan MY. Triacylglycerols: fuelling the hibernating Mycobacterium tuberculosis. Front Cell Infect Microbiol 2019:450. 10.3389/fcimb.2018.00450 PMC6333902 30687647

[31] Laval T, Chaumont L, Demangel C (2021). Not too fat to fight: the emerging role of macrophage fatty acid metabolism in immunity to Mycobacterium tuberculosis. Immunol Rev.

[32] Prabakaran JJ, Efrem B, Araya M, Mebrahtu M, Zeraburuk H, Yemane M (2017). Association of Dietary and serum cholesterol with active pulmonary tuberculosis: a Hospital based study. Int J Curr Microbiol App Sci.

[33] Ahmed N, Amjad I, Malik ZA, Naseer A, Raza M, Imtiaz A et al. Effects of anti-tuberculosis drugs on lipid profile in pulmonary tuberculosis patients. *JDUHS* 2021.

[34] Taparia P, Yadav D, Koolwal S, Mishra S (2015). Study of lipid profile in pulmonary tuberculosis patients and relapse cases in relation with disease severity-A pilot study. Indian J Sci Appl Res.

[35] Jo YS, Han K, Kim D, Yoo JE, Kim Y, Yang B (2021). Relationship between total cholesterol level and tuberculosis risk in a nationwide longitudinal cohort. Sci rep.

[36] Minardi ML, Fato I, Di Gennaro F, Mosti S, Mastrobattista A, Cerva C (2021). Common and rare hematological manifestations and adverse drug events during treatment of active TB: a state of art. Microorganisms.

[37] Balepur SS, Schlossberg D. Hematologic complications of tuberculosis. Tuberculosis and Nontuberculous Mycobacterial Infections 2017:529–39.

[38] Kirwan DE, Chong DL, Friedland JS (2021). Platelet activation and the Immune response to tuberculosis. Front Immunol.

[39] Kullaya V, van der Ven A, Mpagama S, Mmbaga BT, de Groot P, Kibiki G (2018). Platelet-monocyte interaction in Mycobacterium tuberculosis infection. Tuberculosis.

[40] Alamlih L, Albakri M, Ibrahim WH, Khan A, Khan FY (2020). Hematologic characteristics of patients with active pulmonary, extra-pulmonary and disseminated tuberculosis: a study of over six hundred patients. J Tuberc Res.

[41] Cobelens F, Kerkhoff AD (2021). Tuberculosis and anemia—cause or effect?. Environ Health Prev Med.

[42] Gelaw Y, Getaneh Z, Melku M (2021). Anemia as a risk factor for tuberculosis: a systematic review and meta-analysis. Environ Health Prev Med.

[43] Davoudi S, Rasoolinegad M, Younesian M, Hajiabdolbaghi M, Soudbakhsh A, Jafari S (2008). CD4 + cell counts in patients with different clinical manifestations of tuberculosis. Braz J Infect Dis.

[44] Bahir Dar City History., Demographics, Climate and Tourist Attractions [https://typicalethiopian.com/bahir-dar-city-history-demographics-climate-tourist-attractions/].

[45] Bahir Dar City. [https://bahirdar.gov.et/index.php/en/city/people].

[46] Thakar M, Angira F, Pattanapanyasat K, Wu AH, O’Gorman M, Zeng H (2017). CD4 lymphocyte enumeration and hemoglobin assessment aid for priority decisions: a multisite evaluation of the BD FACSPresto™ system. open AIDS J.

[47] Nuttall FQ (2015). Body mass index: obesity, BMI, and health: a critical review. Nutr Today.

[48] A healthy lifestyle. : WHO recommendations. Available at https://www.who.int/europe/news-room/fact-sheets/item/a-healthy-lifestyle---who-recommendations.

[49] WHO. Haemoglobin concentrations for the diagnosis of anaemia and assessment of severity. In.: World Health Organization; 2011.

[50] Body mass index - BMI. [https://www.euro.who.int/en/health-topics/disease-prevention/nutrition/a-healthy-lifestyle/body-mass-index-bmi].

[51] Lichtman MA, Kaushansky K, Prchal JT, Levi MM, Burns LJ, Armitage JO. Williams Manual of Hematology: Table of Normal Values. In: *Williams Manual of Hematology, 9e* edn. New York, NY: McGraw-Hill Education; 2017.

[52] Mulu W, Abera B, Mekonnen Z, Adem Y, Yimer M, Zenebe Y (2017). Haematological and CD4 + T cells reference ranges in healthy adult populations in Gojjam zones in Amhara region, Ethiopia. PLoS ONE.

[53] Patient education. : High cholesterol and lipid treatment options (Beyond the Basics)

[54] Creatinine. blood test [https://www.ucsfhealth.org/medical-tests/creatinine-blood-test#:~:text=Normal%20Results,person’s%20size%20and%20muscle%20mass.].

[55] Abebe M, Melku M, Enawgaw B, Birhan W, Deressa T, Terefe B (2018). Reference intervals of routine clinical chemistry parameters among apparently healthy young adults in Amhara National Regional State, Ethiopia. PLoS ONE.

[56] Fantahun M, Kebede A, Yenew B, Gemechu T, Mamuye Y, Tadesse M (2019). Diagnostic accuracy of Xpert MTB/RIF assay and non-molecular methods for the diagnosis of tuberculosis lymphadenitis. PLoS ONE.

[57] Shetty D, Vyas D (2022). Combination method for the diagnosis of tuberculous lymphadenitis in high burden settings. Surg Exp Pathol.

[58] Hasan Z, Cliff JM, Dockrell HM, Jamil B, Irfan M, Ashraf M (2009). CCL2 responses to Mycobacterium tuberculosis are associated with disease severity in tuberculosis. PLoS ONE.

[59] May RM, Anderson RM. Epidemiology and genetics in the coevolution of parasites and hosts. *Proceedings of the Royal society of London Series B Biological sciences* 1983, 219(1216):281–313. 10.1098/rspb.1983.0075 6139816

[60] Atomsa D, Abebe G, Sewunet T. Immunological markers and hematological parameters among newly diagnosed tuberculosis patients at Jimma University Specialized Hospital. *EJHS* 2014, 24(4):311–318. 10.4314/ejhs.v24i4.6 PMC4248030 25489195

[61] Al-Aska A, Al-Anazi A, Al-Subaei S, Al-Hedaithy M, Barry M, Somily A (2011). CD4 + T-lymphopenia in HIV negative tuberculous patients at King Khalid University Hospital in Riyadh, Saudi Arabia. Eur J Med Res.

[62] Kotlyarov S (2022). High-density lipoproteins: a role in inflammation in COPD. Int J Mol Sci.

[63] Sakamuri RM, Price DN, Lee M, Cho SN, Barry CE, Via LE (2013). Association of lipoarabinomannan with high density lipoprotein in blood: implications for diagnostics. Tuberculosis.

[64] Chidambaram V, Zhou L, Ruelas Castillo J, Kumar A, Ayeh SK, Gupte A (2021). Higher serum cholesterol levels are associated with reduced systemic inflammation and mortality during tuberculosis treatment independent of body mass index. Front cardiovasc med.

[65] Renshaw AA, Gould EW (2013). Thrombocytosis is associated with Mycobacterium tuberculosis infection and positive acid-fast stains in granulomas. Am J Clin Pathol.

[66] Barzegari S, Afshari M, Movahednia M, Moosazadeh M (2019). Prevalence of anemia among patients with tuberculosis: a systematic review and meta-analysis. Indian J Tuberc.

[67] de Mendonca EB, Schmaltz CA, Sant’Anna FM, Vizzoni AG, Mendes-de-Almeida DP, de Oliveira (2021). Anemia in tuberculosis cases: a biomarker of severity?. PLoS ONE.

[68] Baluku JB, Mayinja E, Mugabe P, Ntabadde K, Olum R, Bongomin F. Prevalence of anaemia and associated factors among people with pulmonary tuberculosis in Uganda. Epidemiol Infect 2022:1–19. 10.1017/S0950268822000103 PMC8888272 35022106

[69] Nagu TJ, Spiegelman D, Hertzmark E, Aboud S, Makani J, Matee MI (2014). Anemia at the initiation of tuberculosis therapy is associated with delayed sputum conversion among pulmonary tuberculosis patients in Dar-es-Salaam, Tanzania. PLoS ONE.

[70] Deniz O, Gumus S, Yaman H, Ciftci F, Ors F, Cakir E (2007). Serum total cholesterol, HDL-C and LDL-C concentrations significantly correlate with the radiological extent of disease and the degree of smear positivity in patients with pulmonary tuberculosis. Clin Biochem.

[71] Diriba G, Kebede A, Yaregal Z, Getahun M, Tadesse M, Meaza A (2017). Performance of Mycobacterium Growth Indicator Tube BACTEC 960 with Lowenstein–Jensen method for diagnosis of Mycobacterium tuberculosis at Ethiopian National Tuberculosis Reference Laboratory, Addis Ababa, Ethiopia. BMC Res Notes.

[72] Mekonnen D, Derbie A, Mihret A, Yimer SA, Tønjum T, Gelaw B (2021). Lipid droplets and the transcriptome of Mycobacterium tuberculosis from direct sputa: a literature review. Lipids Health Dis.

